# Role of CD36 in Palmitic Acid Lipotoxicity in Neuro-2a Neuroblastoma Cells

**DOI:** 10.3390/biom11111567

**Published:** 2021-10-22

**Authors:** C. J. Urso, Heping Zhou

**Affiliations:** Department of Biological Sciences, Seton Hall University, 400 South Orange Avenue, South Orange, NJ 07079, USA; ursocj@shu.edu

**Keywords:** fatty acid uptake, palmitic acid, cell cycle, CD36, lipotoxicity, saturated fatty acids

## Abstract

Elevated level of palmitic acid (PA), a long-chain saturated fatty acid (SFA), is lipotoxic to many different types of cells including Neuro-2a (N2a) neuroblastoma cells. CD36 is a multifunctional membrane glycoprotein that acts as a fatty acid translocase (FAT) facilitating the transport of long-chain free fatty acids (FFAs) into cells, serves a fatty acid (FA) sensing function in areas including taste buds and the proximal gut, and acts as a scavenger receptor that binds to many ligands, including FAs, collagen, oxidized low-density lipoproteins, and anionic phospholipids. However, the involvement of CD36 in FA uptake and PA lipotoxicity in N2a cells remains unclear. In this study, we examined FA uptake in BSA- and PA-treated N2a cells and investigated the involvement of CD36 in FA uptake and PA lipotoxicity in N2a cells. Our data showed that PA treatment promoted FA uptake in N2a cells, and that treatment with sulfo-N-succinimidyl oleate (SSO), a CD36 inhibitor, significantly decreased FA uptake in BSA- and PA-treated N2a cells, and ameliorated PA-induced decrease of cell viability, decrease of diploid cells, and increase of tetraploid cells. We also found that CD36 knockdown significantly decreased FA uptake in both BSA- and PA-treated cells as compared to their corresponding wild-type controls, and dramatically attenuated PA-induced cell cycle defects in N2a cells. Our data suggest that CD36 may play a critical role in FA uptake and PA lipotoxicity in N2a cells. CD36 may therefore represent a regulatory target against pathologies caused by excess FAs.

## 1. Introduction

Circulating levels of free fatty acids (FFAs) are reportedly elevated in obese subjects [1,2], overweight/obese subjects with diabetes mellitus [3], and obese non-alcoholic fatty liver disease (NAFLD) patients [3,4]. Excessive level of palmitic acid (PA), a long-chain saturated fatty acid (SFA), has been shown to induce lipotoxicity in many types of cells including skeletal muscles, liver cells, neuronal cells, neuroblastoma, and microglia cells [5,6,7,8,9,10,11]. There have been studies showing that PA induces oxidative stress in rat cortical cells [12], bovine endometrial cells [13], and H9C2 cardiomyocytes [14] as well as endoplasmic reticulum (ER) stress in different cells including H9C2 cardiomyocytes [14], skeletal muscle cells [14,15], pancreatic β cells [16], and hypothalamic neurons [17] while N-acetyl cysteine, an antioxidant, and 4-phenylbutyrate, an inhibitor of ER stress, are not able to alleviate PA-induced decrease of cell viability in neuroblastoma Neuro-2a (N2a) cells [9].

While there are reports that long-chain FFAs may diffuse across the plasma membrane [18], protein-mediated transport of long-chain FFAs across the plasma membrane represents an important mechanism to bring long-chain FFAs into cells [19,20]. Several long-chain fatty acid transporters have been identified, including fatty acid transport proteins (FATPs), plasma membrane-associated fatty acid-binding protein (FABPpm), and cluster of differentiation 36/fatty acid translocase (CD36/FAT) [20]. After entry into cells, FAs are then converted into fatty acyl-CoA by fatty acyl CoA synthetase. Fatty acyl-CoA may then be catabolized to generate energy or anabolized to produce a range of molecules such as second messengers, hormones, and diacylglycerols [21].

CD36 is a multifunctional membrane glycoprotein expressed in a wide range of cells. It serves a FA sensing function in areas including taste buds and the proximal gut and acts as a scavenger receptor that binds to many ligands, including FAs, collagen, oxidized low-density lipoproteins, thrombospondin, amyloid β, and anionic phospholipids [22,23]. CD36 has also been identified as one of the efflux transporters of amyloid β proteins in the cerebral vessels [24]. Moreover, CD36 acts as a FAT facilitating the transport of long-chain FFAs into cells [25]. CD36 expression is increased in the brains of senescence-accelerated mouse-prone (SAMP) 8 mice as compared with that of the control senescence-accelerated mouse-resistant (SAMR) 1 mice [24]. CD36 deficiency alleviates diabetic cardiomyopathy and atherosclerosis and protects against diet-induced obesity, intramuscular lipid deposition, and oxidative stress [26]. Mice lacking CD36 have impaired FA uptake into the muscle, heart, and adipose tissue with excess FAs delivered to the liver leading to steatosis [27].

We have previously reported that PA, the most abundant SFA present in the diet and in serum [28], decreases the viability and increases the death of N2a cells as compared to BSA-treated control [9]. However, the role of CD36 in FA uptake in N2a cells remains unclear, and the mechanisms by which PA induces lipotoxicity in N2a cells have not been defined. We hypothesize that CD36 plays a critical role in mediating FA uptake and PA lipotoxicity in N2a cells. In this study, we first investigated FA uptake in BSA- and PA-treated N2a cells, and then examined whether inhibition of CD36 by its irreversible inhibitor, sulfo-N-succinimidyl oleate (SSO), and CD36 knockdown by its shRNA affected FA uptake and PA lipotoxicity in N2a cells.

## 2. Materials and Methods

### 2.1. Reagents

PA and fatty acid-free bovine serum albumin (BSA) were purchased from Sigma-Aldrich (St. Louis, MO, USA). BSA-conjugated PA was prepared as previously described [9]. PA was dissolved in 100% ethanol at 400 mM and then conjugated with 13.4% BSA at 2.5:1 molar ratio. Conjugated PA was then filtered, aliquoted, and stored at −80 °C. Sulfo-N-succinimidyl oleate (SSO) was purchased from Cayman Chemical (Ann Arbor, MI, USA) and dissolved in DMSO.

### 2.2. Cell Culture

Murine Neuro-2a (N2A) neuroblastoma cells were purchased from American Type Culture Collection (ATCC; Manassas, VA, USA). These cells were cultured in Eagle’s minimum essential media (EMEM) (Lonza; Walkersville, MD, USA) supplemented with 10% (*v*/*v*) fetal bovine serum (FBS) (Invitrogen; Carlsbad, CA, USA) and 1% (*v*/*v*) penicillin-streptomycin (Sigma-Aldrich; St. Louis, MO, USA) and maintained in a humidified environment with 5% CO_2_ at 37 °C. All experiments were conducted with cells of fewer than 20 passages.

### 2.3. Establishment of N2a Cells Stably Transfected with CD36 shRNA

In order to examine whether CD36 knockdown affected FA uptake, N2a cells were stably transfected with MISSION^®^ CD36 shRNA (TRCN0000066518) with MISSION^®^ pLKO.1-puro non-mammalian shRNA plasmid as control from Sigma-Aldrich (St. Louis, MO, USA). Stable transfection of N2a cells using Lipofectamine^TM^ 3000 (ThermoFisher Scientific; Waltham, MA, USA) was conducted following manufacturer’s protocol. Briefly, N2a cells were seeded in a 96-well tissue culture plate at 1 × 10^4^ cells/well and incubated overnight. On the following day, 100 ng of CD36 shRNA plasmid or non-mammalian shRNA control plasmid was diluted in 5 µL Opti-MEM^TM^ media, combined with 0.2 µL P3000™ Reagent, then added 0.15 μL Lipofectamine^TM^ 3000 reagent diluted in 5 μL Opti-MEM^TM^ media, and incubated at room temperature for 10 min. This DNA-lipid complex was then added to N2a cells in EMEM with 10% FBS, incubated for another 24 h, passaged at 1:300 into 96-well plates, and then selected for puromycin resistance in complete media with 5 µg/mL puromycin. The selective media was refreshed every 3–4 days. After 2 weeks of selection, the large healthy colonies were then selected to expand in selective media.

### 2.4. BODIPY^TM^ FL C12 Uptake Assay by Flow Cytometry

To examine FA uptake in N2a cells, BODIPY^TM^ FL C12 (ThermoFisher Scientific; Waltham, MA, USA), a fluorescent FA analog, was used. BODIPY^TM^ FL C12 was deemed to resemble the length of C16 FA such as PA since the BODIPY^TM^ FL fluorophore contributes an additional four-carbon length of acyl chain while BODIPY^TM^ FL C16 would resemble the length of C20 FA. Furthermore, the complex lipid product profile of ^3^H-PA most closely resembles that of BODIPY^TM^ FL C12 based on metabolic tracing performed in zebrafish [29]. Therefore, BODIPY^TM^ FL C12 was deemed as a suitable fluorescent PA analog. BODIPY^TM^ FL C12 uptake assay by flow cytometry was performed as described previously [10]. Briefly, N2a cells were incubated with 15 μM BODIPY^TM^ FL C12 in serum-free media for 30 min at 37 °C. Following 30 min incubation, cells were then collected by trypsinization, resuspended in ice-cold D-PBS, and immediately analyzed by flow cytometry. Flow cytometry events were then gated, and data were analyzed using FlowJo10 (FlowJo; Ashland, OR, USA).

### 2.5. BODIPY^TM^ FL C12 Uptake Assay by Confocal Microscopy

To visualize FA uptake under confocal microscopy, the fluorescent PA analog BODIPY^TM^ FL C12 was used and the uptake assay was performed as described previously [10]. Briefly, N2a cells were treated with BSA or 200 µM PA for 6 h, then incubated with 15 μM BODIPY^TM^ FL C12 for 30 min, washed with PBS, and fixed with 4% paraformaldehyde for 20 min at 4 °C. Cells were then washed with PBS again and stained with 1 μg/mL DAPI for 10 min, and then visualized under FluoView FV1000 confocal microscope (Olympus; Center Valley, PA, USA).

### 2.6. RNA Isolation

Total RNA from N2a cells was isolated using the TRIzol reagent (Sigma-Aldrich; St Louis, MO, USA) following the manufacturer’s instructions. The prepared RNA samples were dissolved in RNase-free water and stored at −80 °C.

### 2.7. Semi-Quantitative Reverse Transcriptase-Polymerase Chain Reaction (RT-PCR) Assay

To assess the effects of PA on CD36 expression, RT-PCR was conducted as described before [29,30,31]. Briefly, cDNA was synthesized from 1 µg of total RNA using oligo (dT)12–18 primer and Moloney murine leukemia virus (M-MLV) reverse transcriptase (Promega, Madison, WI), and then used to detect mouse CD36 and β-actin, a house-keeping gene, by PCR amplification. Appropriate sense and antisense primers specific for mouse CD36 and β-actin were synthesized by Eurofins Genomics (Huntsville, AL, USA). The forward (F) and reverse (R) primers for CD36 were 5′-CTCACTGGAGGAAACTGCTATC-3′ (F) and 5′-CTCCAGAGAGGGAGAGACTTAATA-3′ (R). The forward (F) and reverse (R) primers for β-actin were 5′-AGCCATGTACGTAGCCATCC-3′ (F) and 5′-CTCTCAGCTGTGGTGGTGAA-3′ (R). PCR reactions were conducted in a final volume of 20 µL containing 1 μL of cDNA, 1X PCR buffer, 0.2 μM of each forward and reverse primer, 0.2 mM of dNTPs, and 0.5 unit of Taq DNA polymerase (Applied Biosystems; Foster City, CA, USA) [29,30,31]. The reaction was heated to 94 °C for 5 min, followed by denaturation at 94 °C for 30 s, annealing at 57 °C for 30 s, and extension at 72 °C for 30 s for 31 cycles and 21 cycles for CD36 and β-actin respectively. After the final cycle, a 7-min extension step at 72 °C was included. PCR products were then resolved by gel electrophoresis on 2.0% agarose gel and the gel image was recorded using the FluorChem system (Protein Sample; San Jose, CA, USA). The band intensities of CD36 were digitized using VisionWorks^TM^ LS software (UVP; Upland, CA, USA) and normalized against that of β-actin in the same sample.

### 2.8. MTT (3-[4,5-Dimethylthiazol-2-yl]-2,5 Diphenyl Tetrazolium Bromide) Assay

To assess the effects of PA on cell viability, 3-[4,5-dimethylthiazol-2-yl]-2,5-diphenyltetrazolium bromide (MTT; Sigma-Aldrich; St. Louis, MO, USA) assay was used based on the principle that actively respiring cells convert MTT to insoluble purple formazan. MTT assay was performed as previously described [9]. Briefly, cells were seeded in 96-well plates at 1.0 × 10^4^ cells/well. After overnight incubation, cells were treated with 200 µM PA or BSA for 24 h. At 2 h prior to the end of treatment, 10 µL of 5 mg/mL MTT was added into each well and incubated for another 2 h to allow sufficient conversion of MTT into formazan precipitates, which were then solubilized in 100 µL of isopropanol with 0.04 M HCl. Absorbance was then read at 570 nm on the VarioSkan Lux microplate reader (ThermoFisher Scientific; Waltham, MA, USA) with absorbance at 650 nm as a reference to correct for nonspecific background absorption. Relative cell viability was calculated as the net absorbance of treated cells divided by the net absorbance of BSA-treated control cells.

### 2.9. Cell Cycle Analysis

To assess the effects of PA on the cell cycle of N2a cells, cell cycle analysis was performed by flow cytometry following staining with 4′,6-diamidino-2-phenylindole (DAPI). Cells were seeded in 12-well plates at 1.0 × 10^5^ cells/well. After overnight incubation, cells were treated with 200 µM PA or BSA in serum-free media for 6 h. At the end of treatment, cells were collected by trypsinization, rinsed with D-PBS, and then fixed in 70% ethanol on ice for at least 1 h. Ethanol-fixed cells were then spun down, rinsed with D-PBS, resuspended in D-PBS, and then incubated with 1 μg/mL DAPI staining solution for 30 min in the dark on ice. DAPI fluorescence was then analyzed by flow cytometry on the MACSQuant Analyzer 10 flow cytometer (Miltenyi Biotec; Auburn, CA, USA). Flow cytometry events were then gated, and data were analyzed using FlowJo10 (FlowJo; Ashland, OR, USA).

### 2.10. Detection of Cell Surface Expression of CD36 by Immunostaining and Flow Cytometry

In order to confirm shRNA knockdown of CD36, N2a cells were stained with APC-Vio^®^ 770-conjugated anti-mouse CD36 antibody (Miltenyl Biotec Inc.; Auburn, CA, USA) for cell surface expression of CD36 and examined by flow cytometry following the manufacturer’s instructions. Wild-type N2a cells, non-mammalian control plasmid-transfected cells, and CD36 shRNA-transfected cells were collected, resuspended at 0.5 × 10^6^ cells per 100 ul of FACS staining buffer (PBS with 0.5% BSA and 2 mM EDTA), and incubated with APC-Vio^®^ 770-conjugated anti-mouse CD36 antibody at 1:50 dilution for 15 min at 4 °C. After labeling with CD36 antibody, cells were washed and resuspended in FACS buffer and analyzed by flow cytometry. Flow cytometry events were then gated, and data were analyzed using FlowJo10 (FlowJo; Ashland, OR, USA).

### 2.11. Bicinchoninic acid (BCA) Protein Assay

In order to determine protein concentrations in cell lysates, BCA protein assay kit (ThermoFisher Scientific; Waltham, MA, USA) was used following the manufacturer’s instructions. Briefly, 10 µL of each albumin standard or 1:10 diluted protein sample was mixed with 200 µL BCA working reagent in a 96-well plate, covered, and incubated at 37 °C for 30 min. The plate was then read on the VarioSkan Lux microplate reader (ThermoFisher Scientific; Waltham, MA, USA) at 562 nm.

### 2.12. Western Blot Analysis

To examine the protein level of CD36, cell lysates were prepared from wild-type N2a cells and N2a cells stably transfected with CD36 shRNA. Cells were grown in complete media to 80% confluency in P100 cell culture Petri dishes and then lifted with a cell scraper. Collected cells were spun down, washed twice with ice-cold D-PBS, resuspended, and lysed with 1x RIPA buffer (Sigma-Aldrich; St. Louis, MO, USA) containing 1X protease inhibitor cocktail (Sigma-Aldrich; St. Louis, MO, USA) on ice for 30 min. The lysates were then sonicated and centrifuged for 15 min at 14,000 rpm at 4 °C. The supernatants containing the soluble protein fractions were transferred to new microcentrifuge tubes and labeled as RIPA-S. The pellets were rinsed with RIPA buffer and then resuspended in PBS with 10% SDS and 1x proteinase inhibitor cocktail, sonicated, and centrifuged for 15 min at 14,000 rpm at 4 °C. The supernatants were transferred to new microcentrifuge tubes and labeled as RIPA-R. 30 µg of RIPA-S and 10 µg of RIPA-R proteins samples were then mixed with an equal volume of 2x Laemmli sample buffer, boiled, separated by sodium dodecyl sulfate-polyacrylamide gel electrophoresis (SDS-PAGE), and transferred to polyvinylidene difluoride (PVDF) membranes [30]. The PVDF membranes were blocked with SuperBlock (ThermoFisher Scientific; Waltham, MA, USA) for 1 h at room temperature, and then incubated with anti-CD36 antibody (ThermoFisher Scientific; Waltham, MA, USA) overnight at 4 °C. After washing with TBST three times, the PVDF membrane blots were incubated with anti-rabbit IgG-HRP for 1 h at room temperature (ProteinSimple; St. Jose, CA, USA), washed with TBST three times, developed with Clarity^Max^ ECL substrate (Bio-Rad; Hercules, CA, USA), and imaged under FluorChem E (ProteinSimple; St. Jose, CA, USA). Following imaging, the PVDF membrane blots were stripped with 0.2 M glycine, 0.1% SDS, and 1% Tween-20 for 20 min, washed twice with TBST, blocked with SuperBlock for 1 h, and then probed with anti-Caveolin-1 antibody (Cell Signaling Technology; Danvers, MA, USA). After washing with TBST three times, the PVDF membrane blots were incubated with anti-rabbit IgG-HRP for 1 h at room temperature (ProteinSimple; St. Jose, CA, USA), washed three times, developed with Clarity^Max^ ECL substrate, and imaged under FluorChem E. After imaging, the PVDF membrane blots were stripped again and then re-probed with anti-actin antibody (Sigma-Aldrich; St. Louis, MO, USA) overnight at 4 °C followed by detection with anti-rabbit IgG-HRP, rinsing, development with Clarity^Max^ ECL substrate, and imaging under FluorChem E. Band intensities were quantitated using Image Studio Lite software (LI-COR Biosciences; Lincoln, NE, USA).

### 2.13. Statistical Analysis

Data were analyzed using GraphPad Prism 6 (GraphPad Software; San Diego, CA, USA). Two-way ANOVA followed by Tukey’s multiple comparison test was used to determine statistical significance. *p* < 0.05 was considered statistically significant.

## 3. Results

### 3.1. Effects of PA Treatment on CD36 Expression

**Figure 1:**
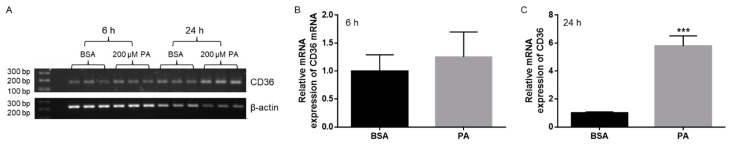
Relative mRNA expression of CD36 in N2a cells following treatment with 200 µM PA or BSA for 6 h and 24 h using semi-quantitative RT-PCR. (**A**) Agarose gel images of CD36 and β-actin representative of three independent experiments. (**B**) Relative CD36 mRNA expression in N2a cells following treatment with BSA or PA for 6 h. (**C**) Relative CD36 mRNA expression in N2a cells following treatment with BSA or PA for 24 h. Data presented (mean ± SD) were representative of three independent experiments. Statistical analysis was performed using *t*-test. *** *p* < 0.001 vs. BSA control.

Previously we reported that 200 µM PA induced a significant decrease in N2a cell viability at 24 h post-treatment, but not at 6 h post-treatment [9]. In order to examine whether CD36-mediated FA uptake was required for PA lipotoxicity, we first investigated the effects of PA treatment on CD36 expression. N2a cells were treated with 200 µM PA or BSA for 6 h or 24 h, and the expression of CD36 in these cells was measured by semi-quantitative RT-PCR. The relative mRNA expression level of CD36 following treatment with PA for 6 h was comparable to that in BSA-treated cells (Figure 1A,B). In contrast, the relative mRNA expression level of CD36 following treatment with PA for 24 h was significantly higher than that in BSA-treated cells (Figure 1A,C).

### 3.2. Time- and Dose-Dependent Effects of PA on BODIPY^TM^ FL C12 Uptake in N2a Cells

**Figure 2:**
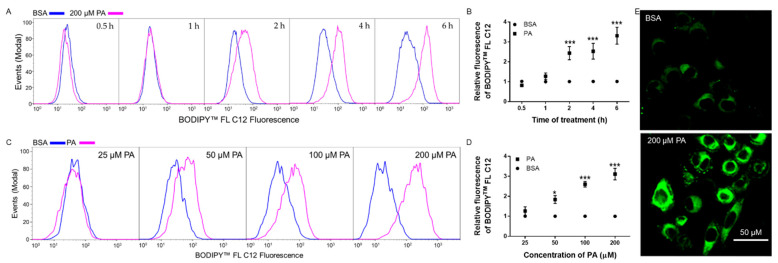
Time- and dose-dependent effects of PA treatment on BODIPY^TM^ FL C12 uptake in N2a cells. (**A**,**B**) N2A cells were treated with 200 µM PA or BSA for 0.5, 1, 2, 4, or 6 h, and then incubated with BODIPY^TM^ FL C12 for 0.5 h. BODIPY^TM^ FL C12 uptake was then measured by flow cytometry with representative histograms shown in (**A**) and quantitated data shown in (**B**–**D**) N2A cells were treated with different concentrations of PA or BSA control for 6 h, and BODIPY^TM^ FL C12 uptake was then measured by flow cytometry with representative histograms shown in (**C**) and quantitated data shown in (**D**,**E**). BODIPY^TM^ FL C12 fluorescence in N2a cells following 6 h treatment with BSA or 200 µM PA visualized under confocal microscopy. Data presented were representative of three independent experiments. Data (Mean ± SEM) were analyzed using two-way ANOVA followed by Tukey’s multiple comparison test. * *p* < 0.05 vs. corresponding BSA control; *** *p* < 0.001 vs. corresponding BSA control.

We next examined the time-dependent effects of PA treatment on FA uptake in N2a. N2a cells were treated with 200 µM PA or BSA control for 0.5, 1, 2, 4, or 6 h, and FA uptake was analyzed by incubation with 15 μM 4,4-Difluoro-5,7-Dimethyl-4-Bora-3a,4a-Diaza-s-Indacene-3-Dodecanoic Acid (BODIPY^TM^ FL C12), a widely used fluorescent long-chain FA analog resembling the acyl chain length of PA. BODIPY™ FL C12 is a well-established fluorescent FA analog that has been used to study FA uptake in various models including white adipose tissue [31,32], multiple cell lines [33,34], and human placenta explants [35]. After 30 min incubation with BODIPY™ FL C12, its fluorescence intensity was measured by flow cytometry. Compared to time-paired BSA controls, the amount of BODIPY^TM^ FL C12 uptake in N2a cells was significantly increased following 2 htreatment with PA and continued to increase at 4 h and 6 h post-treatment (Figure 2A,B). Therefore 6 h PA treatment was selected for further investigation. Taken together with mRNA expression data, these data suggest that more rapid dynamic cellular processes other than CD36 expression regulation may be responsible for PA-induced potentiation of FA uptake at up to 6 h post-treatment. We also examined the dose-dependent effects of PA on FA uptake in N2a cells. N2a cells were treated with different concentrations of PA for 6 h and BODIPY^TM^ FL C12 uptake was then examined. PA started to significantly increase BODIPY^TM^ FL C12 uptake at 50 µM. As the concentration of PA increased to 200 µM, BODIPY^TM^ FL C12 uptake continued to increase in N2a cells (Figure 2C,D). We also examined BODIPY^TM^ FL C12 uptake in cells treated with 200 µM PA or BSA for 6 h under confocal microscopy. As shown in Figure 2E, 200 µM PA significantly increased the amount of BODIPY^TM^ FL C12 fluorescence as compared to BSA control. These data confirmed that treatment with PA significantly enhanced FA uptake in N2a cells, therefore PA treatment may promote its own uptake in N2a cells.

### 3.3. Sulfo-N-Succinimidyl Oleate (SSO) Decreased BODIPY^TM^ FL C12 Uptake in N2a Cells

**Figure 3:**
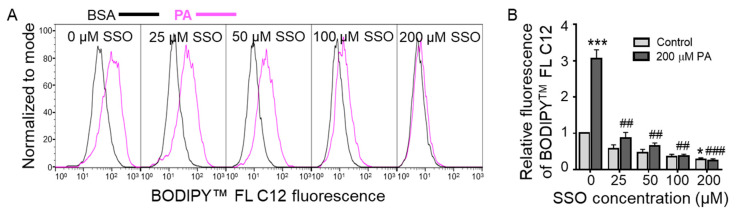
BODIPY^TM^ FL C12 uptake in N2a cells pre-treated with sulfo-N-succinimidyl oleate (SSO) for 1 h followed by treatment with BSA or PA for 6 h. N2a cells were pre-treated with different concentrations of SSO for 1 h followed by treatment with 200 µM PA or BSA for 6 h, and FA uptake in these cells was determined by BODIPY^TM^ FL C12 via flow cytometry with representative histograms shown in (**A**) and quantitated data shown in (**B**). Data presented were representative of three independent experiments. Data (Mean ± SEM) were analyzed using two-way ANOVA followed by Tukey’s multiple comparison test. * *p* < 0.05 vs. BSA control; *** *p* < 0.001 vs. BSA control; ## *p* < 0.01 vs. 200 µM PA; ### *p* < 0.001 vs. 200 µM PA.

To examine the involvement of CD36 in FA uptake in N2a cells, cells were pre-treated with different concentrations of *Sulfo-N*-succinimidyl oleate (SSO), a well-known irreversible inhibitor that effectively and completely blocks CD36-mediated FA uptake, for 1 h followed by treatment with BSA or 200 µM PA for 6 h and then BODIPY^TM^ FL C12 uptake in these cells was examined by flow cytometry. As shown in Figure 3, pretreatment with SSO at a concentration as low as 25 µM significantly decreased PA-induced increase of BODIPY^TM^ FL C12 uptake. As the SSO concentration increased to 200 µM, BODIPY^TM^ FL C12 uptake in PA-treated cells was further inhibited. At 200 µM, SSO also significantly decreased BODIPY^TM^ FL C12 uptake in BSA-treated cells. These data suggest that CD36 may play a key role in BODIPY^TM^ FL C12 uptake in both BSA- and PA-treated cells and that CD36-mediated PA uptake may contribute to PA lipotoxicity in N2a cells.

### 3.4. SSO Attenuated PA-Induced Decrease in N2a Cell Viability

**Figure 4:**
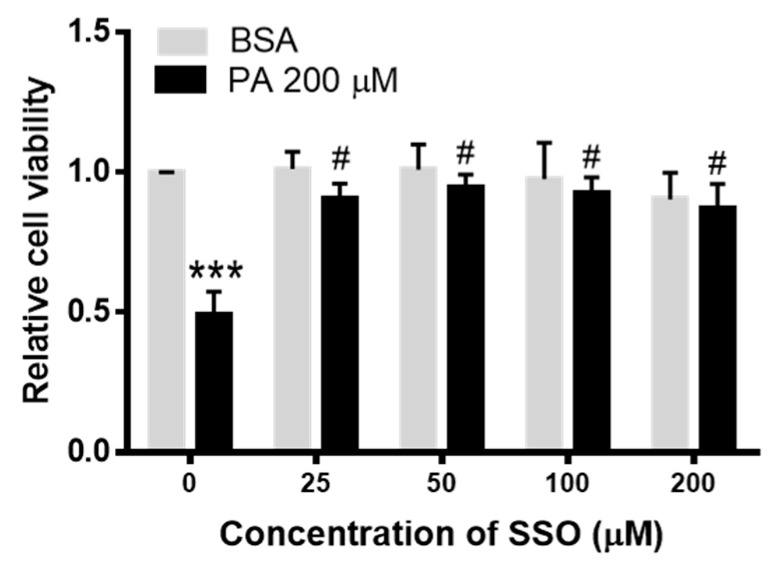
Cell viability analysis of N2a cells pre-treated with sulfo-N-succinimidyl oleate (SSO) for 1 h followed by treatment with BSA or PA for 24 h. N2a cells were pre-treated with different concentrations of SSO for 1 h followed by treatment with 200 µM PA or BSA for 6 h, and cell viability was assessed by MTT assay. Data presented were representative of three independent experiments. Data (Mean ± SEM) were analyzed using two-way ANOVA followed by Tukey’s multiple comparison test. *** *p* < 0.001 vs. BSA control; # *p* < 0.05 vs. 200 µM PA.

We previously showed that 200 µM PA did not decrease cell viability at 6 h, but significantly decreased cell viability at 24 h [9], therefore we examined whether SSO inhibition of CD36 could attenuate PA-induced decrease of N2a cell viability. N2a cells were pre-treated with different concentrations of SSO for 1 h followed by treatment BSA or 200 µM PA for 24 h, and cell viability was assessed by MTT assay. As shown in Figure 4, SSO at 25 µM started to significantly attenuate PA-induced decrease of cell viability and continued to abolish PA-induced decrease of cell viability at higher concentrations tested.

### 3.5. SSO Attenuated PA-Induced Cell Cycle Defects in N2a Cells

**Figure 5:**
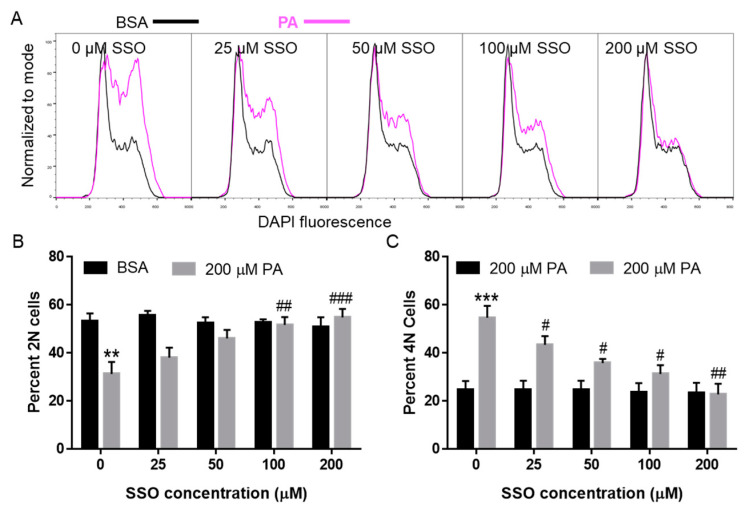
Cell cycle analysis of N2a cells following treatment with 200 µM PA or BSA together with different concentrations of SSO. N2a cells were pre-treated with different concentrations of SSO for 1 h followed by incubation with 200 µM PA or BSA for 6 h, their cell cycle profile was determined by DAPI via flow cytometry with representative histograms shown in (**A**), quantitated percent 2N cells shown in (**B**), and quantitated percent 4N cells shown in (**C**). Data presented (Mean ± SEM) were representative of three independent experiments. Data were analyzed using two-way ANOVA followed by Tukey’s multiple comparison test. ** *p* < 0.01 vs. BSA control; *** *p* < 0.001 vs. BSA control; # *p* < 0.05 vs. 200 µM PA; ## *p* < 0.01 vs. 200 µM PA; ### *p* < 0.001 vs. 200 µM PA.

We also examined whether SSO inhibition of CD36 could abolish PA-induced cell cycle defects. N2a cells were pre-treated with different concentrations of SSO for 1 h followed by treatment with BSA or 200 µM PA for 6 h, and then the cell cycle profile was examined by DAPI staining followed by flow cytometry analysis. As shown in Figure 5, 200 µM PA significantly decreased the percent of 2N cells and increased the percent of 4N cells at 6 h post-treatment as compared to the BSA control. 25 µM SSO significantly attenuated PA-induced increase of 4N cells. At 100 µM, SSO significantly attenuated PA-induced decrease of 2N cells and diminished PA-induced increase of 4N cells. At 200 µM, SSO completely abolished PA-induced increase of 4N cells and decrease of 2N cells, restoring the cell cycle profile that is comparable to BSA-treated control cells (Figure 5). These data suggest that CD36 may be involved in PA-induced accumulation of 4N cells.

### 3.6. CD36 Knockdown Attenuated BODIPY^TM^ FL C12 Uptake

**Figure 6:**
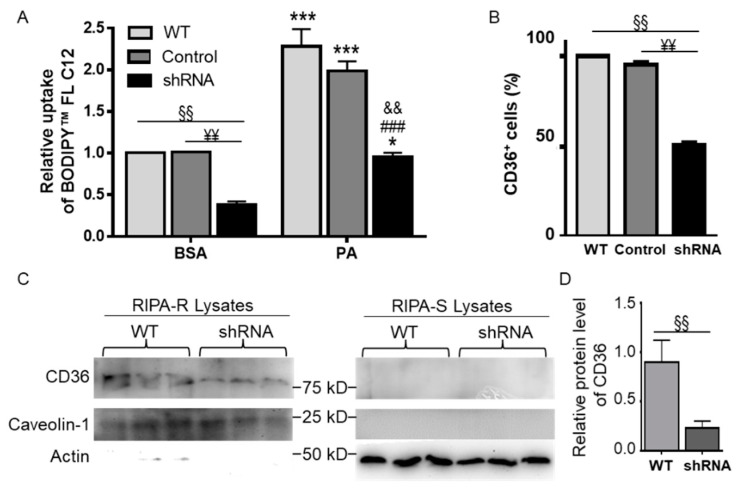
BODIPY^TM^ FL C12 uptake in N2a cells stably transfected with CD36 shRNA. (**A**) Wildtype (WT), non-mammalian control shRNA-transfected (control), and CD36 shRNA-transfected cells (shRNA) were treated with BSA or 200 µM PA for 6 h. BODIPY^TM^ FL C12 uptake was then examined by flow cytometry. Data were analyzed using two-way ANOVA followed by Tukey’s multiple comparison test. (**B**) Detection of CD36 surface expression in WT, control, and CD36 shRNA cells by immunostaining with APC-Vio^®^ 770-conjugated anti-mouse CD36 followed by flow cytometry. Data were analyzed using t-test. (**C**,**D**) Detection of CD36 protein by Western blot assay. WT and CD36 shRNA cells were lysed in RIPA buffer, and the soluble fraction was collected as RIPA-S. The RIPA-insoluble fractions (RIPA-R) were solubilized by 10% SDS. Both fractions were subjected to Western blot analysis and detected by CD36, Caveolin-1, and actin antibodies with Western blot images shown in (**C**)and quantitated data shown in (**D**). Data were analyzed using t-test. ¥¥ *p* < 0.01 within comparison groups; §§ *p* < 0.01 within comparison groups; * *p* < 0.05 vs. corresponding BSA-treated control cells; *** *p* < 0.001 vs. corresponding BSA-treated control cells; ### *p* < 0.001 vs. 200 µM PA-treated WT cells; && *p* < 0.01 vs. 200 µM PA-treated non-mammalian control shRNA-transfected cells.

To confirm the involvement of CD36 in FA uptake and PA lipotoxicity, N2a cells were stably transfected with CD36 shRNA or non-mammalian shRNA control plasmid. Wild-type (WT), non-mammalian control shRNA-transfected, and CD36 shRNA-transfected N2a cells were treated with BSA or 200 µM PA for 6 h, and then BODIPY™ FL C12 uptake was assessed by flow cytometry. As shown in Figure 6A, BODIPY™ FL C12 uptake in BSA- or PA-treated CD36 shRNA-transfected cells was significantly lower than that in corresponding BSA- or PA-treated WT or control cells. As PA treatment significantly increased BODIPY™ FL C12 uptake in WT and non-mammalian shRNA-transfected control cells, PA treatment also dramatically enhanced BODIPY™ FL C12 uptake in CD36 shRNA-transfected cells as compared to the corresponding BSA-treated cells. These data confirmed that CD36 may be important for FA uptake in both BSA- and PA-treated cells.

To confirm CD36 knockdown by shRNA, the surface expression of CD36 in WT, control, and CD36 shRNA-transfected N2a cells was examined by immunostaining with fluorescence-conjugated CD36 antibody and flow cytometry analysis. The percent of CD36^+^ cells in CD36 shRNA-transfected cells was significantly lower than that in WT and control cells (Figure 6B). Total protein level of CD36 was also examined by Western blot assay. CD36 protein was not detected in RIPA-soluble (RIPA-S) fractions but was detected in RIPA-resistant (RIPA-R) membrane fractions solubilized with 10% SDS (Figure 6C). Actin was used as a marker for RIPA-soluble fractions. Calveolin-1, a membrane protein marker, was used for normalization of the relative protein level of CD36 in RIPA-R fractions. As shown in Figure 6C,D, the relative protein level of CD36 in shRNA-transfected cells was significantly lower than that in WT cells.

### 3.7. CD36 Knockdown Attenuated PA-Induced Cell Cycle Defects

**Figure 7:**
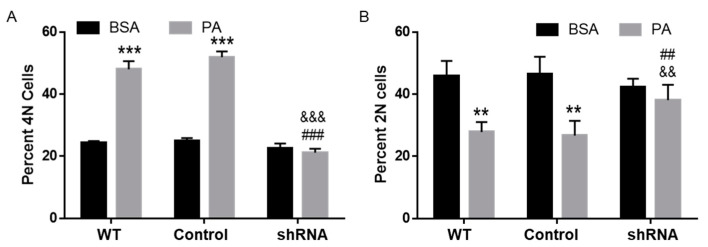
Cell cycle analysis of N2a cells stably transfected with CD36 shRNA. N2a cells stably transfected with CD36 shRNA or non-mammalian shRNA were treated with BSA or 200 µM PA for 6 h with WT cells as naïve control. Their cell cycle profiles were then analyzed by flow cytometry with quantitated 4N data shown in (**A**) and quantitated 2N data shown in (**B**). Data presented (Mean ± SEM) were representative of three independent experiments. Data were analyzed using two-way ANOVA followed by Tukey’s multiple comparison test. ** *p* < 0.01 vs. corresponding BSA-treated cells; *** *p* < 0.001 vs. corresponding BSA-treated cells; ## *p* < 0.01 vs. 200 µM PA-treated control cells; ### *p* < 0.001 vs. 200 µM PA-treated WT cells; && *p* < 0.01 vs. 200 µM PA-treated control-transfected cells; &&& *p* < 0.001 vs. 200 µM PA-treated control-transfected cells.

Since inhibition of CD36 with SSO abrogated the lipotoxic effects of PA on N2a cell cycle progression, we next examined cell cycle progression in N2a cells with CD36 knockdown. To this end, WT, control-transfected, and CD36 shRNA-transfected cells were treated with BSA or 200 µM PA for 6 h, and their cell cycle profiles were determined using DAPI staining followed by flow cytometry analysis. While PA treatment significantly increased the percent of 4N cells in WT and control-transfected cells, shRNA knockdown of CD36 abolished PA-induced increase of 4N cells (Figure 7A). While PA treatment significantly decreased the percent of 2N cells in WT and control-transfected cells, shRNA knockdown of CD36 abolished PA-induced decrease of 2N cells (Figure 7B). These data suggest that CD36 may play a key role in PA lipotoxicity.

## 4. Discussion

We have previously reported that 24 h treatment with 200 µM PA significantly decreases cell viability and induces cell death in N2a cells [9]. While several FA transport proteins are expressed in N2a cells, this study was designed to examine the involvement of CD36 in FA uptake and PA lipotoxicity in N2a cells. It has been previously reported that uptake of FA analogs, such as 15-(*p*-iodophenyl)-3-(R,S)-methyl pentadecanoic acid and 15-(p-iodophenyl)pentadecanoic acid, in the heart, skeletal muscle, and adipose tissue is significantly decreased in CD36 knockout mice [36]. In our study, treatment with SSO, a well-known irreversible CD36 inhibitor, not only blocked BODIPY™ FL C12 uptake in BSA- and PA-treated cells but also abolished PA-induced decrease in cell viability and accumulation of 4N cells, and that knockdown of CD36 decreased BODIPY™ FL C12 uptake in both BSA- and PA-treated cells as compared to the corresponding WT and control-transfected cells and ameliorated PA-induced cell cycle defects. These results suggest that CD36 may play an important role in FA uptake and PA lipotoxicity in N2a cells.

While simple diffusion has been posited as a mechanism for FA uptake [18], our data contributed to the growing evidence that uptake of long-chain FA is primarily mediated by protein transporters [20]. CD36 is widely expressed in many types of cells including hepatocytes, adipocytes, microvascular endothelial cells, cardiomyocytes, astrocytes, microglia, and neurons [37,38,39]. In this study, we used BODIPY^TM^ FL C12 to examine FA uptake for several reasons. First, BODIPY^TM^ FL C12 was deemed to resemble the length of C16 FA such as PA since the BODIPY^TM^ FL fluorophore contributes an additional four-carbon length of acyl chain. Second, the complex lipid product profile of ^3^H-PA most closely resembles that of BODIPY^TM^ FL C12 based on metabolic tracing performed in zebrafish [29]. Third, as a widely-used PA analog that is fluorescent in both aqueous and lipid environments, BODIPY^TM^ FL C12 has been used to trace the movement of FA across the cell layers of living explants of human term placenta [35], and has been shown to be incorporated into LD in hepatocytes [34]. Therefore, this study employed BODIPY^TM^ FL C12 to examine FA uptake. While the focus of this paper was on the involvement of CD36 in FA uptake and PA lipotoxicity, it remains of importance to further examine the incorporation of BODIPY^TM^ FL C12 after entering N2a cells.

The maturation and transportation of CD36 are regulated by post-translational modifications, including phosphorylation, ubiquitination, glycosylation, and palmitoylation [26]. Palmitoylation of CD36 has been shown to target CD36 to the plasma membrane and facilitate fatty acid uptake in adipose cells [40]. PA oversupply has also been shown to increase the translocation of CD36 to sarcolemma in cardiac [41,42] and skeletal muscles [43], and to enhance lipid uptake in heart and skeletal muscles [42,43]. Therefore, it is likely that PA treatment may increase the palmitoylation of CD36 in N2a cells, thereby increasing CD36-mediated FA uptake in N2a cells. Future studies on post-translational modification of CD36 such as palmitoylation in PA-treated cells as compared to BSA-treated cells may help to shed light on the regulatory mechanisms of CD36 following PA treatment in N2a cells.

Studies have suggested that CD36-mediated FA transport involves its reversible trafficking between intracellular membrane compartments and plasma membrane [42,44] and its dynamic distribution in its localization in non-lipid raft membranes and lipid raft membranes [45]. Lipid rafts are known to contain high concentrations of PA, cholesterol, and sphingosine. It has been shown that the membrane lipid composition and physicochemical properties of lipid rafts are subjected to modifications by exogenous FAs [46]. For example, depletion of PA disrupts the lipid raft domains [47]. Furthermore, CD36-mediated FA uptake is significantly enhanced when its association with lipid rafts is increased [45]. It is likely that PA treatment may enhance the association of CD36 with lipid rafts, thereby augmenting CD36-mediated FA uptake in N2a cells. Therefore, future studies should examine the incorporation of PA and localization of CD36 in PA-treated cells as compared to BSA-treated cells.

Our data also showed that PA treatment increased BODIPY™ FL C12 uptake in WT, control-transfected, and CD36 shRNA-transfected cells as compared to their corresponding BSA-treated cells, which suggests that PA treatment may augment the uptake of FA into N2a cells. In contrast, SSO inhibition of CD36 was able to diminish BODIPY™ FL C12 uptake in both BSA- and PA-treated cells to minimal levels. As an irreversible CD36 inhibitor, SSO was able to inhibit CD36 function regardless of its localization in lipid rafts and its post-translational modification status. However, shRNA knockdown of CD36 only significantly decreased CD36 protein, but had no effects on post-translational regulation of CD36 function. Potential regulation of CD36 palmitoylation and lipid raft localization by PA may be able to account for increased FA uptake in CD36 knockdown cells.

CD36 has been shown to play an important role in hypothalamic FA sensing and food intake regulation [48]. CD36 gene polymorphism −31118 G > A (rs1761667) is associated with overweight and obesity [49]. CD36 deficiency prevents obesity-associated cardiac steatosis and insulin resistance [50]. It has been implicated in dysregulated lipid metabolism in diabetic cardiomyopathy [42], Alzheimer’s disease [51,52], and demyelinating disorders such as multiple sclerosis [53]. Besides acting as a FA transporter, CD36 also acts as a receptor for multiple ligands with signal transduction capabilities. For example, it has been shown to initiate signaling by Src kinase partners and regulating Ca^2+^ activation of phospholipases [27]. Therefore, it is worthwhile to investigate whether and how CD36-mediated signaling may contribute to PA lipotoxicity in N2a cells.

Lipids are known to play important roles in brain development, neurogenesis, synaptogenesis, myelin sheath formation, and signal transduction [54]. Alteration of lipid homeostasis is associated with various disorders and pathologies including Alzheimer’s and Parkinson’s disease [55,56,57]. Lipotoxicity has been implicated in various neurological pathologies including diabetic neuropathy and cognitive decline [58,59]. Our studies suggest that CD36 may represent a regulatory target against pathologies caused by excess FAs, and that understanding the mechanisms that mediate and regulate CD36 function will help to understand the mechanisms of FA accumulation and neuropathologies in obese and metabolic syndrome patients.

## 5. Conclusions

In summary, our study showed that inhibition of CD36 with SSO significantly decreased BODIPY™ FL C12 uptake in BSA- and PA-treated cells, and blocked PA-induced decrease of cell viability, decrease of 2N cells and increase of 4N cells. Our data also showed that knockdown of CD36 by shRNA significantly decreased FA uptake in BSA- and PA-treated cells as compared to the corresponding WT or control-transfected cells, and abolished PA-induced decrease of 2N cells, and increase of 4N cells. These results demonstrate that CD36 is a critical mediator of FA uptake and that CD36-mediated PA uptake may play an important role in PA lipotoxicity in N2a cells. Our data also showed that PA treatment enhanced BODIPY™ FL C12 uptake in WT, control-transfected, and CD36 knockdown cells. It remains to be determined whether PA-induced increase in FA uptake could be attributed to PA regulation of post-translational modification and localization of CD36.

## Data Availability

Not applicable.

## References

[1] Arner P., Ryden M. (2015). Fatty Acids, Obesity and Insulin Resistance. Obes. Facts.

[2] Rodriguez-Pacheco F., Gutierrez-Repiso C., Garcia-Serrano S., Alaminos-Castillo M.A., Ho-Plagaro A., Valdes S., Garcia-Arnes J., Gonzalo M., Andrade R.J., Moreno-Ruiz F.J. (2016). The pro-/anti-inflammatory effects of different fatty acids on visceral adipocytes are partially mediated by GPR120. Eur. J. Nutr..

[3] Zhang J., Zhao Y., Xu C., Hong Y., Lu H., Wu J., Chen Y. (2014). Association between serum free fatty acid levels and nonalcoholic fatty liver disease: A cross-sectional study. Sci. Rep..

[4] Feng R., Luo C., Li C., Du S., Okekunle A.P., Li Y., Chen Y., Zi T., Niu Y. (2017). Free fatty acids profile among lean, overweight and obese non-alcoholic fatty liver disease patients: A case-control study. Lipids Health Dis..

[5] Capel F., Acquaviva C., Pitois E., Laillet B., Rigaudiere J.P., Jouve C., Pouyet C., Gladine C., Comte B., Vianey Saban C. (2015). DHA at nutritional doses restores insulin sensitivity in skeletal muscle by preventing lipotoxicity and inflammation. J. Nutr. Biochem..

[6] Cheon H.G., Cho Y.S. (2014). Protection of palmitic acid-mediated lipotoxicity by arachidonic acid via channeling of palmitic acid into triglycerides in C_2_C_12_. J. Biomed. Sci..

[7] Chen X., Li L., Liu X., Luo R., Liao G., Liu J., Cheng J., Lu Y., Chen Y. (2018). Oleic acid protects saturated fatty acid mediated lipotoxicity in hepatocytes and rat of non-alcoholic steatohepatitis. Life Sci..

[8] Kwon B., Lee H.K., Querfurth H.W. (2014). Oleate prevents palmitate-induced mitochondrial dysfunction, insulin resistance and inflammatory signaling in neuronal cells. Biochim. Biophys. Acta.

[9] Urso C.J., Zhou H. (2021). Differential Effects of Unsaturated Fatty Acids and Saturated Fatty Acids in Lipotoxicity and Neutral Lipid Accumulation in Neuro-2a Cells. Biomed. J. Sci. Tech. Res..

[10] Urso C.J., Zhou H. (2021). Palmitic Acid Lipotoxicity in Microglia Cells Is Ameliorated by Unsaturated Fatty Acids. Int. J. Mol. Sci..

[11] Zhou H., Urso C.J., Jadeja V. (2020). Saturated Fatty Acids in Obesity-Associated Inflammation. J. Inflamm. Res..

[12] Almaguel F.G., Liu J.W., Pacheco F.J., Casiano C.A., De Leon M. (2009). Activation and reversal of lipotoxicity in PC12 and rat cortical cells following exposure to palmitic acid. J. Neurosci. Res..

[13] Li P., Li L., Zhang C., Cheng X., Zhang Y., Guo Y., Long M., Yang S., He J. (2019). Palmitic Acid and beta-Hydroxybutyrate Induce Inflammatory Responses in Bovine Endometrial Cells by Activating Oxidative Stress-Mediated NF-kappaB Signaling. Molecules.

[14] Yang L., Guan G., Lei L., Liu J., Cao L., Wang X. (2019). Oxidative and endoplasmic reticulum stresses are involved in palmitic acid-induced H_9_c_2_ cell apoptosis. Biosci. Rep..

[15] Yuzefovych L., Wilson G., Rachek L. (2010). Different effects of oleate vs. palmitate on mitochondrial function, apoptosis, and insulin signaling in L6 skeletal muscle cells: Role of oxidative stress. Am. J. Physiol. Endocrinol. Metab..

[16] Suzuki E., Matsuda T., Kawamoto T., Takahashi H., Mieda Y., Matsuura Y., Takai T., Kanno A., Koyanagi-Kimura M., Asahara S.I. (2018). Docosahexaenoic Acid Reduces Palmitic Acid-Induced Endoplasmic Reticulum Stress in Pancreatic Beta Cells. Kobe J. Med. Sci..

[17] Mayer C.M., Belsham D.D. (2010). Palmitate attenuates insulin signaling and induces endoplasmic reticulum stress and apoptosis in hypothalamic neurons: Rescue of resistance and apoptosis through adenosine 5’ monophosphate-activated protein kinase activation. Endocrinology.

[18] Kamp F., Hamilton J.A. (2006). How fatty acids of different chain length enter and leave cells by free diffusion. Prostaglandins Leukot. Essent. Fatty Acids.

[19] Jay A.G., Hamilton J.A. (2018). The enigmatic membrane fatty acid transporter CD36: New insights into fatty acid binding and their effects on uptake of oxidized LDL. Prostaglandins Leukot. Essent. Fatty Acids.

[20] Schwenk R.W., Holloway G.P., Luiken J.J., Bonen A., Glatz J.F. (2010). Fatty acid transport across the cell membrane: Regulation by fatty acid transporters. Prostaglandins Leukot. Essent. Fatty Acids.

[21] Alves-Bezerra M., Cohen D.E. (2017). Triglyceride Metabolism in the Liver. Compr. Physiol..

[22] Park Y.M. (2014). CD36, a scavenger receptor implicated in atherosclerosis. Exp. Mol. Med..

[23] Silverstein R.L., Febbraio M. (2009). CD36, a scavenger receptor involved in immunity, metabolism, angiogenesis, and behavior. Sci. Signal..

[24] Wu B., Ueno M., Kusaka T., Miki T., Nagai Y., Nakagawa T., Kanenishi K., Hosomi N., Sakamoto H. (2013). CD36 expression in the brains of SAMP8. Arch. Gerontol. Geriatr..

[25] Verpoorten S., Sfyri P., Scully D., Mitchell R., Tzimou A., Mougios V., Patel K., Matsakas A. (2020). Loss of CD36 protects against diet-induced obesity but results in impaired muscle stem cell function, delayed muscle regeneration and hepatic steatosis. Acta Physiol..

[26] Shu H., Peng Y., Hang W., Nie J., Zhou N., Wang D.W. (2020). The role of CD36 in cardiovascular disease. Cardiovasc. Res..

[27] Pepino M.Y., Kuda O., Samovski D., Abumrad N.A. (2014). Structure-function of CD36 and importance of fatty acid signal transduction in fat metabolism. Annu. Rev. Nutr..

[28] Baylin A., Kabagambe E.K., Siles X., Campos H. (2002). Adipose tissue biomarkers of fatty acid intake. Am. J. Clin. Nutr..

[29] Quinlivan V.H., Wilson M.H., Ruzicka J., Farber S.A. (2017). An HPLC-CAD/fluorescence lipidomics platform using fluorescent fatty acids as metabolic tracers. J. Lipid Res..

[30] Surriga O., Ortega A., Jadeja V., Bellafronte A., Lasala N., Zhou H. (2009). Altered hepatic inflammatory response in the offspring following prenatal LPS exposure. Immunol. Lett..

[31] Varlamov O., Chu M.P., McGee W.K., Cameron J.L., O’Rourke R.W., Meyer K.A., Bishop C.V., Stouffer R.L., Roberts C.T. (2013). Ovarian cycle-specific regulation of adipose tissue lipid storage by testosterone in female nonhuman primates. Endocrinology.

[32] Salameh A., Daquinag A.C., Staquicini D.I., An Z., Hajjar K.A., Pasqualini R., Arap W., Kolonin M.G. (2016). Prohibitin/annexin 2 interaction regulates fatty acid transport in adipose tissue. JCI Insight.

[33] Ahowesso C., Black P.N., Saini N., Montefusco D., Chekal J., Malosh C., Lindsley C.W., Stauffer S.R., DiRusso C.C. (2015). Chemical inhibition of fatty acid absorption and cellular uptake limits lipotoxic cell death. Biochem. Pharmacol..

[34] Wang H., Wei E., Quiroga A.D., Sun X., Touret N., Lehner R. (2010). Altered lipid droplet dynamics in hepatocytes lacking triacylglycerol hydrolase expression. Mol. Biol. Cell.

[35] Kolahi K., Louey S., Varlamov O., Thornburg K. (2016). Real-Time Tracking of BODIPY-C12 Long-Chain Fatty Acid in Human Term Placenta Reveals Unique Lipid Dynamics in Cytotrophoblast Cells. PLoS ONE.

[36] Coburn C.T., Knapp F.F., Febbraio M., Beets A.L., Silverstein R.L., Abumrad N.A. (2000). Defective uptake and utilization of long chain fatty acids in muscle and adipose tissues of CD36 knockout mice. J. Biol. Chem..

[37] Quintana-Castro R., Aguirre-Maldonado I., Soto-Rodriguez I., Deschamps-Lago R.A., Gruber-Pagola P., Urbina de Larrea Y.K., Juarez-Rivera V.E., Ramos-Manuel L.E., Alexander-Aguilera A. (2020). Cd36 gene expression in adipose and hepatic tissue mediates the lipids accumulation in liver of obese rats with sucrose-induced hepatic steatosis. Prostaglandins Other Lipid Mediat..

[38] Mitchell R.W., Edmundson C.L., Miller D.W., Hatch G.M. (2009). On the mechanism of oleate transport across human brain microvessel endothelial cells. J. Neurochem..

[39] Ioghen O., Chitoiu L., Gherghiceanu M., Ceafalan L.C., Hinescu M.E. (2021). CD36—A novel molecular target in the neurovascular unit. Eur. J. Neurosci..

[40] Wang J., Hao J.W., Wang X., Guo H., Sun H.H., Lai X.Y., Liu L.Y., Zhu M., Wang H.Y., Li Y.F. (2019). DHHC4 and DHHC5 Facilitate Fatty Acid Uptake by Palmitoylating and Targeting CD36 to the Plasma Membrane. Cell Rep..

[41] Zhu B., Li M.Y., Lin Q., Liang Z., Xin Q., Wang M., He Z., Wang X., Wu X., Chen G.G. (2020). Lipid oversupply induces CD36 sarcolemmal translocation via dual modulation of PKCzeta and TBC1D1: An early event prior to insulin resistance. Theranostics.

[42] Glatz J.F.C., Luiken J., Nabben M. (2020). CD36 (SR-B2) as a Target to Treat Lipid Overload-Induced Cardiac Dysfunction. J. Lipid Atheroscler..

[43] Chabowski A., Gorski J., Luiken J.J., Glatz J.F., Bonen A. (2007). Evidence for concerted action of FAT/CD36 and FABPpm to increase fatty acid transport across the plasma membrane. Prostaglandins Leukot. Essent. Fatty Acids.

[44] Luiken J.J., Chanda D., Nabben M., Neumann D., Glatz J.F. (2016). Post-translational modifications of CD36 (SR-B2): Implications for regulation of myocellular fatty acid uptake. Biochim. Biophys. Acta.

[45] Ehehalt R., Sparla R., Kulaksiz H., Herrmann T., Fullekrug J., Stremmel W. (2008). Uptake of long chain fatty acids is regulated by dynamic interaction of FAT/CD36 with cholesterol/sphingolipid enriched microdomains (lipid rafts). BMC Cell Biol..

[46] Anderson R.G., Jacobson K. (2002). A role for lipid shells in targeting proteins to caveolae, rafts, and other lipid domains. Science.

[47] Ventura R., Mordec K., Waszczuk J., Wang Z., Lai J., Fridlib M., Buckley D., Kemble G., Heuer T.S. (2015). Inhibition of de novo Palmitate Synthesis by Fatty Acid Synthase Induces Apoptosis in Tumor Cells by Remodeling Cell Membranes, Inhibiting Signaling Pathways, and Reprogramming Gene Expression. EBioMedicine.

[48] Moulle V.S., Le Foll C., Philippe E., Kassis N., Rouch C., Marsollier N., Bui L.C., Guissard C., Dairou J., Lorsignol A. (2013). Fatty acid transporter CD36 mediates hypothalamic effect of fatty acids on food intake in rats. PLoS ONE.

[49] Enciso-Ramirez M., Reyes-Castillo Z., Llamas-Covarrubias M.A., Guerrero L., Lopez-Espinoza A., Valdes-Miramontes E.H. (2020). CD36 gene polymorphism -31118 G > A (rs1761667) is associated with overweight and obesity but not with fat preferences in Mexican children. Int. J. Vitam. Nutr. Res..

[50] Gharib M., Tao H., Fungwe T.V., Hajri T. (2016). Cluster Differentiating 36 (CD36) Deficiency Attenuates Obesity-Associated Oxidative Stress in the Heart. PLoS ONE.

[51] Ricciarelli R., D’Abramo C., Zingg J.M., Giliberto L., Markesbery W., Azzi A., Marinari U.M., Pronzato M.A., Tabaton M. (2004). CD36 overexpression in human brain correlates with beta-amyloid deposition but not with Alzheimer’s disease. Free. Radic. Biol. Med..

[52] Sery O., Janoutova J., Ewerlingova L., Halova A., Lochman J., Janout V., Khan N.A., Balcar V.J. (2017). CD36 gene polymorphism is associated with Alzheimer’s disease. Biochimie.

[53] Grajchen E., Wouters E., van de Haterd B., Haidar M., Hardonniere K., Dierckx T., Van Broeckhoven J., Erens C., Hendrix S., Kerdine-Romer S. (2020). CD36-mediated uptake of myelin debris by macrophages and microglia reduces neuroinflammation. J. Neuroinflamm..

[54] Hussain G., Anwar H., Rasul A., Imran A., Qasim M., Zafar S., Imran M., Kamran S.K.S., Aziz N., Razzaq A. (2020). Lipids as biomarkers of brain disorders. Crit. Rev. Food Sci. Nutr..

[55] Adibhatla R.M., Hatcher J.F. (2008). Phospholipase A(2), reactive oxygen species, and lipid peroxidation in CNS pathologies. BMB Rep..

[56] Chew H., Solomon V.A., Fonteh A.N. (2020). Involvement of Lipids in Alzheimer’s Disease Pathology and Potential Therapies. Front. Physiol..

[57] Alecu I., Bennett S.A.L. (2019). Dysregulated Lipid Metabolism and Its Role in alpha-Synucleinopathy in Parkinson’s Disease. Front. Neurosci..

[58] Opazo-Rios L., Mas S., Marin-Royo G., Mezzano S., Gomez-Guerrero C., Moreno J.A., Egido J. (2020). Lipotoxicity and Diabetic Nephropathy: Novel Mechanistic Insights and Therapeutic Opportunities. Int. J. Mol. Sci..

[59] Hidalgo-Lanussa O., Baez-Jurado E., Echeverria V., Ashraf G.M., Sahebkar A., Garcia-Segura L.M., Melcangi R.C., Barreto G.E. (2020). Lipotoxicity, neuroinflammation, glial cells and oestrogenic compounds. J. Neuroendocrinol..

